# Azido­[1,2-bis­(diphenyl­phosphan­yl)ethane-κ^2^ *P*,*P*′](η^5^-inden­yl)ruthenium(II)

**DOI:** 10.1107/S1600536810053006

**Published:** 2010-12-24

**Authors:** Hui-Ling Sung, Hsiu-Ling Hsu, Ting-Shen Kuo

**Affiliations:** a Department of Mathematics and Science (Pre-college), National Taiwan Normal University, Taiwan; b Department of Chemical and Materials Engineering, Lunghwa University of Science and Technology, Taiwan; c Department of Chemistry, National Taiwan Normal University, 11677, Taiwan

## Abstract

Facile ligand substitution is observed when the ruthenium chloride complex [Ru(η^5^-C_9_H_7_)Cl(dppe)] (dppe is diphenylphosphanyl ethane) is treated with NaN_3_ in refluxing ethanol, yielding the title compound, [Ru(η^5^-C_9_H_7_)(N_3_)(dppe)] or [Ru(C_9_H_7_)(N_3_)(C_26_H_24_P_2_)]. The Ru(II) atom has a typical piano-stool coordination. The Ru—P bond lengths are 2.284 (2) and 2.235 (2) Å. NMR and MS analyses are in agreement with the structure of the title compound.

## Related literature

For the synthesis of the title compound, see: Singh *et al.* (2005 ▶). For the chemistry of organic azides, see: Labbe (1969 ▶); Patai (1971 ▶). For metal–azido complexes, see: Dori & Ziolo (1973 ▶); Frühauf (1997 ▶). Organic azides are particularly important for the synthesis of heterocyclic compounds by reaction with 1,3-dipole compounds, see: Padwa (1976 ▶). Metal–azido complexes have been reported to produce tetra­zolates by reaction with nitrile and isonitriles, see: Beck & Schropp (1975 ▶); Ellis & Purcell (1982 ▶); Fehlhammer & Dahl (1972 ▶); Paul & Nag (1987 ▶); Treichel *et al.* (1971 ▶).
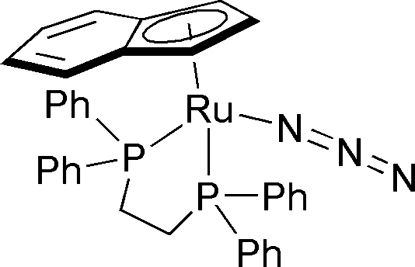


## Experimental

### 

#### Crystal data

[Ru(C_9_H_7_)(N_3_)(C_26_H_24_P_2_)]
*M* *_r_* = 656.64
Monoclinic, 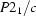

*a* = 11.331 (6) Å
*b* = 14.567 (9) Å
*c* = 17.873 (11) Å
β = 96.015 (19)°
*V* = 2934 (3) Å^3^
*Z* = 4
Mo *K*α radiation
μ = 0.67 mm^−1^
*T* = 200 K
0.22 × 0.10 × 0.04 mm

#### Data collection

Bruker Kappa APEXII CCD area-detector diffractometer
Absorption correction: multi-scan (*SADABS*; Bruker, 2005 ▶) *T*_min_ = 0.866, *T*_max_ = 0.974
20374 measured reflections
5167 independent reflections
2438 reflections with *I* > 2σ(*I*)
*R*_int_ = 0.155

#### Refinement

*R*[*F* ^2^ > 2σ(*F* ^2^)] = 0.053
*wR*(*F* ^2^) = 0.096
*S* = 0.75
5167 reflections
370 parameters
H-atom parameters constrained
Δρ_max_ = 0.54 e Å^−3^
Δρ_min_ = −0.53 e Å^−3^

### 

Data collection: *APEX2* (Bruker, 2007 ▶); cell refinement: *SAINT* (Bruker, 2007 ▶); data reduction: *SAINT*; program(s) used to solve structure: *SHELXS97* (Sheldrick, 2008 ▶); program(s) used to refine structure: *SHELXL97* (Sheldrick, 2008 ▶); molecular graphics: *ORTEP-3 for Windows* (Farrugia, 1997 ▶); software used to prepare material for publication: *WinGX* (Farrugia, 1999) ▶.

## Supplementary Material

Crystal structure: contains datablocks global, I. DOI: 10.1107/S1600536810053006/rn2075sup1.cif

Structure factors: contains datablocks I. DOI: 10.1107/S1600536810053006/rn2075Isup2.hkl

Additional supplementary materials:  crystallographic information; 3D view; checkCIF report

## Figures and Tables

**Fig. 1.**
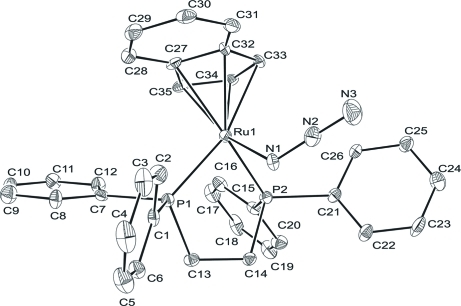
View of the title compound showing displacement ellipsoids at the 30% probability level. H atoms are omitted for clarity.

## supplementary crystallographic information

### Comment

Organic azides are particularly important for synthesizing heterocyclic compounds by reaction with 1,3-dipole compounds (Padwa, 1976). Metal azido complexes have been reported to produce tetrazolates by reaction with nitrile (Paul & Nag, 1987; Ellis & Purcell, 1982) and isonitriles (Treichel *et al.*, 1971; Beck & Schropp, 1975; Fehlhammer & Dahl, 1972).

Treatment of the complex [Ru(η^5^-C_9_H_7_)Cl(dppe)] with sodium azide in ethanol afforded the title compound [Ru(η^5^-C_9_H_7_)N_3_(dppe)] (Figure 1). In the crystal structure of the title compound, the azide groups are almost linear [N(3)—N(2)—N(1)=175.5 (8)°] and are coordinated to Ru with an Ru—N—N angle of 119.0 (5)°.

### Experimental

To a solution of [Ru(η^5^-C_9_H_7_)Cl(dppe)] (0.1 g, 0.154 mmol) in ethanol (30 ml), an excess of NaN_3_ (0.05 g, 0.769 mmol) was added. The mixture was heated to reflux for 4 h and cooled to room temperature. The solvent was dried under vacuum and 10 ml of CH_2_Cl_2_ was added to the residue. The product was dissolved in CH_2_Cl_2_ and other salts such as NaN_3_ and NaCl precipitated. After filtration, the solvent of the mixture was concentrated to about 5 ml. The residue was then slowly added to 40 ml of vigorously stirred diethyl ether. The orange precipitate thus formed was filtered off, washed with diethyl ether and hexane and dried under vacuum to give the title compound [Ru(η^5^-C_9_H_7_)N_3_(dppe)] (0.08 g, 0.122 mmol) in 79% yield. The orange crystals of the title compound for X-ray structure analysis were obtained by slow diffusion of diethyl ether into a CH_2_Cl_2_ solution at room temperature for 3 days. Spectroscopic analysis: ^1^H NMR (CDCl_3_, 298 K, δ, p.p.m.): 7.44—7.20 (m, 24H, 20H of Ph group, 4H of indenyl ring), 4.91 (t, 1H, ^3^J_H—H_= 1.30 Hz, H of indenyl ring), 5.51 (d, 2H, ^3^J_H—H_= 2.15 Hz, H of indenyl ring), 2.38, 2.29 (m, 4H, 2CH_2_ of dppe). ^31^P{^1^H} NMR (CDCl_3_, 298 K, δ, p.p.m.): 85.3. ^13^C{^1^H} NMR (CDCl_3_, 298 K, δ, p.p.m.): 141—108 (Ph and indenyl group), 29.2 (t, J_C—P_= 22.64 Hz, CH_2_ of dppe). HRMS (ESI, m/*z*): 657.1 (*M*^+^), 615.3 (*M*^+^—N_3_). Anal. Calcd for C_35_H_31_N_3_P_2_Ru: C, 64.02; H, 4.76; N, 6.40. Found: C, 64.16; H, 4.82; N, 6.28.

### Refinement

All H atoms were initially located in a difference map, but were constrained to an idealized geometry. Constrained bond lengths and isotropic displacement parameters: C—H = 0.95 Å and *U*_iso_(H) = 1.2 *U*_eq_(C) for aromatic H atoms, and C—H = 0.99 Å and *U*_iso_(H) = 1.2 *U*_eq_(C) for methylene.

### Crystal data

**Table:**

[Ru(C_9_H_7_)(N_3_)(C_26_H_24_P_2_)]	*F*(000) = 1344
*M**_r_* = 656.64	*D*_x_ = 1.487 Mg m^−^^3^
Monoclinic, *P*2_1_/*c*	Mo *K*α radiation, λ = 0.71073 Å
*a* = 11.331 (6) Å	Cell parameters from 1167 reflections
*b* = 14.567 (9) Å	θ = 2.6–19.4°
*c* = 17.873 (11) Å	µ = 0.67 mm^−^^1^
β = 96.015 (19)°	*T* = 200 K
*V* = 2934 (3) Å^3^	Prism, orange-brown
*Z* = 4	0.22 × 0.10 × 0.04 mm

### Data collection

**Table:**

Bruker Kappa APEXII CCD area-detector diffractometer	5167 independent reflections
Radiation source: fine-focus sealed tube	2438 reflections with *I* > 2σ(*I*)
graphite	*R*_int_ = 0.155
Detector resolution: 9 pixels mm^-1^	θ_max_ = 25.0°, θ_min_ = 1.8°
CCD rotation images, thick slices scans	*h* = −13→10
Absorption correction: multi-scan (*SADABS*; Bruker, 2005)	*k* = −16→17
*T*_min_ = 0.866, *T*_max_ = 0.974	*l* = −21→20
20374 measured reflections	

### Refinement

**Table:**

Refinement on *F*^2^	Primary atom site location: structure-invariant direct methods
Least-squares matrix: full	Secondary atom site location: difference Fourier map
*R*[*F*^2^ > 2σ(*F*^2^)] = 0.053	Hydrogen site location: inferred from neighbouring sites
*wR*(*F*^2^) = 0.096	H-atom parameters constrained
*S* = 0.75	*w* = 1/[σ^2^(*F*_o_^2^) + (0.0072*P*)^2^] where *P* = (*F*_o_^2^ + 2*F*_c_^2^)/3
5167 reflections	(Δ/σ)_max_ = 0.001
370 parameters	Δρ_max_ = 0.54 e Å^−^^3^
0 restraints	Δρ_min_ = −0.53 e Å^−^^3^

### Special details

**Table:**

Geometry. All e.s.d.'s (except the e.s.d. in the dihedral angle between two l.s. planes)are estimated using the full covariance matrix. The cell e.s.d.'s are takeninto account individually in the estimation of e.s.d.'s in distances, anglesand torsion angles; correlations between e.s.d.'s in cell parameters are onlyused when they are defined by crystal symmetry. An approximate (isotropic)treatment of cell e.s.d.'s is used for estimating e.s.d.'s involving l.s. planes.
Refinement. Refinement of *F*^2^ against ALL reflections. The weighted *R*-factor *wR* and goodness of fit *S* are based on *F*^2^, conventional *R*-factors *R* are based on *F*, with *F* set to zero for negative *F*^2^. The threshold expression of *F*^2^ > σ(*F*^2^) is used only for calculating *R*-factors(gt) *etc*. and is not relevant to the choice of reflections for refinement. *R*-factors based on *F*^2^ are statistically about twice as large as those based on *F*, and *R*- factors based on ALL data will be even larger.

### Fractional atomic coordinates and isotropic or equivalent isotropic displacement parameters (Å^2^)

**Table:**

	*x*	*y*	*z*	*U*_iso_*/*U*_eq_	
C1	0.2833 (6)	−0.0987 (4)	0.6432 (4)	0.0325 (18)	
C2	0.2129 (7)	−0.1041 (5)	0.7006 (5)	0.049 (2)	
H2	0.2220	−0.0601	0.7401	0.059*	
C3	0.1277 (7)	−0.1734 (6)	0.7021 (5)	0.059 (3)	
H3	0.0776	−0.1764	0.7415	0.071*	
C4	0.1185 (8)	−0.2362 (6)	0.6459 (6)	0.068 (3)	
H4	0.0606	−0.2834	0.6457	0.082*	
C5	0.1902 (8)	−0.2329 (5)	0.5900 (5)	0.064 (3)	
H5	0.1836	−0.2790	0.5522	0.077*	
C6	0.2728 (6)	−0.1636 (4)	0.5869 (4)	0.043 (2)	
H6	0.3214	−0.1608	0.5467	0.052*	
C7	0.3040 (6)	0.0764 (4)	0.5736 (3)	0.0283 (17)	
C8	0.1933 (6)	0.0576 (5)	0.5384 (4)	0.0391 (18)	
H8	0.1545	0.0022	0.5497	0.047*	
C9	0.1373 (7)	0.1183 (5)	0.4863 (4)	0.051 (2)	
H9	0.0611	0.1040	0.4616	0.062*	
C10	0.1919 (7)	0.1986 (5)	0.4707 (4)	0.042 (2)	
H10	0.1531	0.2403	0.4354	0.050*	
C11	0.3001 (7)	0.2193 (4)	0.5050 (4)	0.042 (2)	
H11	0.3376	0.2751	0.4932	0.050*	
C12	0.3570 (6)	0.1599 (4)	0.5572 (4)	0.040 (2)	
H12	0.4324	0.1758	0.5822	0.048*	
C13	0.5126 (5)	−0.0399 (4)	0.5971 (4)	0.0340 (18)	
H13A	0.4876	−0.0918	0.5633	0.041*	
H13B	0.5410	0.0103	0.5663	0.041*	
C14	0.6120 (5)	−0.0706 (4)	0.6553 (4)	0.0317 (17)	
H14A	0.6859	−0.0807	0.6315	0.038*	
H14B	0.5904	−0.1286	0.6794	0.038*	
C15	0.7216 (6)	0.1045 (4)	0.6777 (4)	0.0324 (18)	
C16	0.6805 (7)	0.1910 (4)	0.6570 (4)	0.039 (2)	
H16	0.6040	0.2091	0.6688	0.047*	
C17	0.7460 (7)	0.2512 (5)	0.6202 (4)	0.055 (2)	
H17	0.7153	0.3103	0.6069	0.066*	
C18	0.8549 (8)	0.2266 (6)	0.6024 (4)	0.057 (3)	
H18	0.9004	0.2688	0.5768	0.069*	
C19	0.9000 (7)	0.1414 (6)	0.6211 (4)	0.058 (2)	
H19	0.9761	0.1244	0.6079	0.070*	
C20	0.8349 (6)	0.0805 (5)	0.6592 (4)	0.045 (2)	
H20	0.8669	0.0219	0.6730	0.053*	
C21	0.7412 (6)	−0.0228 (4)	0.7992 (4)	0.0316 (18)	
C22	0.7937 (6)	−0.1062 (4)	0.7984 (5)	0.046 (2)	
H22	0.7785	−0.1444	0.7554	0.055*	
C23	0.8685 (7)	−0.1366 (5)	0.8588 (5)	0.062 (3)	
H23	0.9070	−0.1943	0.8565	0.075*	
C24	0.8876 (6)	−0.0838 (5)	0.9224 (4)	0.050 (2)	
H24	0.9376	−0.1055	0.9647	0.060*	
C25	0.8347 (6)	−0.0003 (5)	0.9246 (4)	0.043 (2)	
H25	0.8478	0.0368	0.9684	0.052*	
C26	0.7628 (6)	0.0299 (4)	0.8635 (4)	0.041 (2)	
H26	0.7266	0.0886	0.8653	0.049*	
C27	0.3015 (7)	0.1547 (4)	0.7838 (4)	0.0366 (19)	
C28	0.1798 (7)	0.1421 (4)	0.7569 (4)	0.043 (2)	
H28	0.1481	0.1675	0.7101	0.051*	
C29	0.1103 (7)	0.0932 (5)	0.7995 (5)	0.050 (2)	
H29	0.0285	0.0858	0.7826	0.060*	
C30	0.1562 (6)	0.0527 (5)	0.8687 (4)	0.045 (2)	
H30	0.1052	0.0183	0.8970	0.054*	
C31	0.2735 (6)	0.0626 (5)	0.8951 (4)	0.0415 (18)	
H31	0.3040	0.0346	0.9411	0.050*	
C32	0.3488 (6)	0.1149 (4)	0.8534 (4)	0.0294 (17)	
C33	0.4714 (6)	0.1389 (4)	0.8655 (4)	0.0319 (18)	
H33	0.5261	0.1246	0.9116	0.038*	
C34	0.4981 (6)	0.1950 (4)	0.8056 (4)	0.035 (2)	
H34	0.5756	0.2270	0.8028	0.042*	
C35	0.4014 (7)	0.2019 (4)	0.7521 (4)	0.038 (2)	
H35	0.3957	0.2409	0.7058	0.046*	
N1	0.4613 (5)	−0.0796 (3)	0.8004 (3)	0.0340 (16)	
N2	0.4855 (6)	−0.0931 (4)	0.8660 (4)	0.0495 (19)	
N3	0.5048 (7)	−0.1117 (5)	0.9303 (4)	0.082 (3)	
P1	0.38539 (16)	0.00036 (11)	0.64504 (10)	0.0282 (5)	
P2	0.63247 (16)	0.02162 (11)	0.72512 (11)	0.0288 (5)	
Ru1	0.45473 (5)	0.05865 (3)	0.75994 (3)	0.02632 (16)	

### Atomic displacement parameters (Å^2^)

**Table:**

	*U*^11^	*U*^22^	*U*^33^	*U*^12^	*U*^13^	*U*^23^
C1	0.027 (5)	0.030 (4)	0.037 (5)	−0.005 (3)	−0.011 (4)	0.006 (3)
C2	0.043 (6)	0.047 (5)	0.058 (6)	−0.004 (4)	0.001 (5)	−0.003 (4)
C3	0.044 (6)	0.069 (6)	0.062 (7)	−0.017 (5)	−0.007 (5)	0.029 (5)
C4	0.065 (7)	0.052 (6)	0.082 (9)	−0.038 (5)	−0.024 (6)	0.029 (5)
C5	0.082 (8)	0.045 (5)	0.061 (7)	−0.022 (5)	−0.018 (6)	0.005 (5)
C6	0.057 (6)	0.029 (4)	0.041 (6)	−0.003 (4)	−0.009 (4)	−0.001 (4)
C7	0.037 (5)	0.028 (4)	0.021 (4)	0.002 (3)	0.006 (4)	−0.002 (3)
C8	0.031 (5)	0.035 (4)	0.049 (5)	−0.004 (4)	−0.009 (4)	0.008 (4)
C9	0.045 (6)	0.051 (5)	0.057 (6)	−0.001 (4)	0.001 (5)	0.001 (4)
C10	0.047 (6)	0.042 (5)	0.034 (5)	0.017 (4)	−0.003 (4)	0.007 (4)
C11	0.061 (6)	0.024 (4)	0.038 (5)	0.001 (4)	0.001 (5)	0.008 (3)
C12	0.044 (5)	0.030 (4)	0.043 (5)	−0.003 (4)	−0.004 (4)	0.004 (4)
C13	0.032 (5)	0.031 (4)	0.040 (5)	−0.003 (3)	0.007 (4)	−0.006 (3)
C14	0.024 (4)	0.032 (4)	0.038 (5)	0.001 (3)	0.001 (4)	−0.007 (4)
C15	0.025 (5)	0.045 (4)	0.027 (5)	−0.012 (3)	0.006 (4)	−0.006 (3)
C16	0.048 (6)	0.039 (4)	0.030 (5)	−0.014 (4)	0.004 (4)	0.005 (4)
C17	0.049 (6)	0.062 (5)	0.053 (6)	−0.021 (5)	0.001 (5)	0.008 (4)
C18	0.045 (6)	0.079 (6)	0.045 (6)	−0.036 (5)	−0.009 (5)	0.020 (5)
C19	0.020 (5)	0.105 (7)	0.049 (6)	−0.018 (5)	0.006 (4)	−0.003 (5)
C20	0.032 (5)	0.055 (5)	0.048 (5)	−0.004 (4)	0.013 (4)	−0.007 (4)
C21	0.017 (4)	0.035 (4)	0.043 (5)	0.000 (3)	0.004 (4)	0.000 (3)
C22	0.034 (5)	0.032 (4)	0.066 (7)	0.004 (4)	−0.020 (5)	−0.012 (4)
C23	0.054 (6)	0.034 (5)	0.091 (8)	0.020 (4)	−0.027 (6)	−0.008 (5)
C24	0.040 (5)	0.058 (6)	0.048 (6)	−0.011 (4)	−0.010 (4)	−0.002 (4)
C25	0.033 (5)	0.043 (5)	0.051 (6)	0.004 (4)	−0.005 (4)	−0.005 (4)
C26	0.044 (5)	0.036 (4)	0.041 (5)	0.017 (4)	−0.001 (4)	0.001 (4)
C27	0.034 (5)	0.031 (4)	0.046 (6)	0.013 (4)	0.010 (4)	−0.007 (4)
C28	0.040 (6)	0.044 (5)	0.043 (6)	0.008 (4)	−0.001 (5)	−0.004 (4)
C29	0.021 (5)	0.065 (6)	0.062 (7)	0.000 (4)	0.000 (5)	−0.023 (5)
C30	0.035 (5)	0.054 (5)	0.050 (6)	−0.002 (4)	0.020 (4)	−0.012 (5)
C31	0.037 (5)	0.055 (4)	0.033 (5)	−0.001 (4)	0.006 (4)	−0.012 (4)
C32	0.031 (5)	0.037 (4)	0.019 (4)	0.005 (3)	−0.005 (4)	−0.003 (3)
C33	0.030 (5)	0.042 (4)	0.022 (5)	0.000 (3)	−0.006 (4)	−0.009 (3)
C34	0.026 (5)	0.029 (4)	0.050 (6)	−0.004 (3)	0.006 (4)	−0.018 (4)
C35	0.046 (5)	0.018 (4)	0.053 (6)	0.005 (3)	0.017 (5)	−0.002 (3)
N1	0.035 (4)	0.037 (4)	0.028 (4)	−0.001 (3)	−0.006 (3)	0.005 (3)
N2	0.056 (5)	0.036 (4)	0.056 (6)	0.003 (3)	0.003 (5)	0.013 (4)
N3	0.103 (7)	0.076 (5)	0.065 (6)	0.007 (4)	−0.004 (6)	0.023 (5)
P1	0.0299 (12)	0.0245 (10)	0.0298 (12)	0.0000 (8)	0.0008 (10)	0.0009 (8)
P2	0.0272 (12)	0.0289 (9)	0.0303 (13)	−0.0012 (8)	0.0024 (10)	−0.0014 (9)
Ru1	0.0255 (3)	0.0245 (3)	0.0284 (3)	−0.0003 (3)	0.0004 (2)	−0.0020 (3)

### Geometric parameters (Å, °)

**Table:**

C1—C2	1.367 (8)	C19—H19	0.9500
C1—C6	1.376 (8)	C20—H20	0.9500
C1—P1	1.847 (6)	C21—C22	1.354 (8)
C2—C3	1.400 (9)	C21—C26	1.383 (8)
C2—H2	0.9500	C21—P2	1.830 (7)
C3—C4	1.354 (10)	C22—C23	1.374 (9)
C3—H3	0.9500	C22—H22	0.9500
C4—C5	1.354 (10)	C23—C24	1.371 (9)
C4—H4	0.9500	C23—H23	0.9500
C5—C6	1.382 (9)	C24—C25	1.358 (8)
C5—H5	0.9500	C24—H24	0.9500
C6—H6	0.9500	C25—C26	1.365 (9)
C7—C8	1.371 (8)	C25—H25	0.9500
C7—C12	1.400 (8)	C26—H26	0.9500
C7—P1	1.861 (7)	C27—C28	1.423 (9)
C8—C9	1.388 (9)	C27—C32	1.425 (9)
C8—H8	0.9500	C27—C35	1.487 (9)
C9—C10	1.366 (9)	C27—Ru1	2.304 (6)
C9—H9	0.9500	C28—C29	1.354 (9)
C10—C11	1.346 (9)	C28—H28	0.9500
C10—H10	0.9500	C29—C30	1.419 (9)
C11—C12	1.382 (8)	C29—H29	0.9500
C11—H11	0.9500	C30—C31	1.370 (9)
C12—H12	0.9500	C30—H30	0.9500
C13—C14	1.517 (8)	C31—C32	1.412 (8)
C13—P1	1.848 (6)	C31—H31	0.9500
C13—H13A	0.9900	C32—C33	1.426 (8)
C13—H13B	0.9900	C32—Ru1	2.307 (6)
C14—P2	1.832 (6)	C33—C34	1.406 (8)
C14—H14A	0.9900	C33—Ru1	2.210 (6)
C14—H14B	0.9900	C33—H33	1.0000
C15—C16	1.380 (8)	C34—C35	1.380 (9)
C15—C20	1.403 (8)	C34—Ru1	2.184 (6)
C15—P2	1.837 (6)	C34—H34	1.0000
C16—C17	1.362 (8)	C35—Ru1	2.173 (6)
C16—H16	0.9500	C35—H35	1.0000
C17—C18	1.354 (9)	N1—N2	1.192 (8)
C17—H17	0.9500	N1—Ru1	2.139 (5)
C18—C19	1.369 (9)	N2—N3	1.178 (8)
C18—H18	0.9500	P1—Ru1	2.284 (2)
C19—C20	1.379 (8)	P2—Ru1	2.235 (2)
			
C2—C1—C6	119.8 (7)	C28—C27—C32	120.3 (7)
C2—C1—P1	116.4 (5)	C28—C27—C35	132.9 (7)
C6—C1—P1	123.7 (6)	C32—C27—C35	106.8 (7)
C1—C2—C3	120.9 (8)	C28—C27—Ru1	125.5 (5)
C1—C2—H2	119.5	C32—C27—Ru1	72.1 (4)
C3—C2—H2	119.5	C35—C27—Ru1	65.9 (3)
C4—C3—C2	118.2 (8)	C29—C28—C27	118.6 (7)
C4—C3—H3	120.9	C29—C28—H28	120.7
C2—C3—H3	120.9	C27—C28—H28	120.7
C5—C4—C3	121.3 (8)	C28—C29—C30	121.8 (7)
C5—C4—H4	119.4	C28—C29—H29	119.1
C3—C4—H4	119.4	C30—C29—H29	119.1
C4—C5—C6	121.1 (8)	C31—C30—C29	120.7 (7)
C4—C5—H5	119.5	C31—C30—H30	119.7
C6—C5—H5	119.5	C29—C30—H30	119.7
C1—C6—C5	118.7 (7)	C30—C31—C32	119.5 (7)
C1—C6—H6	120.7	C30—C31—H31	120.2
C5—C6—H6	120.7	C32—C31—H31	120.2
C8—C7—C12	118.0 (6)	C31—C32—C27	119.2 (7)
C8—C7—P1	124.0 (5)	C31—C32—C33	133.2 (7)
C12—C7—P1	117.9 (5)	C27—C32—C33	107.6 (6)
C7—C8—C9	120.8 (7)	C31—C32—Ru1	125.4 (4)
C7—C8—H8	119.6	C27—C32—Ru1	71.9 (4)
C9—C8—H8	119.6	C33—C32—Ru1	67.9 (4)
C10—C9—C8	119.8 (8)	C34—C33—C32	108.2 (6)
C10—C9—H9	120.1	C34—C33—Ru1	70.4 (4)
C8—C9—H9	120.1	C32—C33—Ru1	75.4 (4)
C11—C10—C9	120.6 (7)	C34—C33—H33	125.7
C11—C10—H10	119.7	C32—C33—H33	125.7
C9—C10—H10	119.7	Ru1—C33—H33	125.7
C10—C11—C12	120.3 (7)	C35—C34—C33	110.5 (7)
C10—C11—H11	119.8	C35—C34—Ru1	71.1 (4)
C12—C11—H11	119.8	C33—C34—Ru1	72.3 (4)
C11—C12—C7	120.3 (7)	C35—C34—H34	124.7
C11—C12—H12	119.8	C33—C34—H34	124.7
C7—C12—H12	119.8	Ru1—C34—H34	124.7
C14—C13—P1	109.6 (4)	C34—C35—C27	106.5 (7)
C14—C13—H13A	109.8	C34—C35—Ru1	72.0 (4)
P1—C13—H13A	109.8	C27—C35—Ru1	75.5 (3)
C14—C13—H13B	109.8	C34—C35—H35	126.3
P1—C13—H13B	109.8	C27—C35—H35	126.3
H13A—C13—H13B	108.2	Ru1—C35—H35	126.3
C13—C14—P2	106.4 (4)	N2—N1—Ru1	119.0 (5)
C13—C14—H14A	110.4	N3—N2—N1	175.5 (8)
P2—C14—H14A	110.4	C13—P1—C1	105.1 (3)
C13—C14—H14B	110.4	C13—P1—C7	103.0 (3)
P2—C14—H14B	110.4	C1—P1—C7	100.8 (3)
H14A—C14—H14B	108.6	C13—P1—Ru1	108.8 (2)
C16—C15—C20	117.1 (6)	C1—P1—Ru1	117.5 (2)
C16—C15—P2	122.5 (5)	C7—P1—Ru1	119.7 (2)
C20—C15—P2	120.4 (5)	C21—P2—C14	105.1 (3)
C17—C16—C15	122.1 (7)	C21—P2—C15	101.8 (3)
C17—C16—H16	119.0	C14—P2—C15	101.8 (3)
C15—C16—H16	119.0	C21—P2—Ru1	116.2 (2)
C18—C17—C16	119.9 (8)	C14—P2—Ru1	108.2 (2)
C18—C17—H17	120.0	C15—P2—Ru1	121.7 (2)
C16—C17—H17	120.0	N1—Ru1—C35	157.8 (2)
C17—C18—C19	120.6 (7)	N1—Ru1—C34	137.1 (3)
C17—C18—H18	119.7	C35—Ru1—C34	36.9 (2)
C19—C18—H18	119.7	N1—Ru1—C33	102.3 (2)
C18—C19—C20	119.8 (7)	C35—Ru1—C33	63.0 (3)
C18—C19—H19	120.1	C34—Ru1—C33	37.3 (2)
C20—C19—H19	120.1	N1—Ru1—P2	82.33 (15)
C19—C20—C15	120.4 (7)	C35—Ru1—P2	117.78 (19)
C19—C20—H20	119.8	C34—Ru1—P2	98.59 (19)
C15—C20—H20	119.8	C33—Ru1—P2	111.50 (18)
C22—C21—C26	117.9 (7)	N1—Ru1—P1	87.17 (16)
C22—C21—P2	125.0 (6)	C35—Ru1—P1	103.4 (2)
C26—C21—P2	116.9 (5)	C34—Ru1—P1	135.8 (2)
C21—C22—C23	121.1 (7)	C33—Ru1—P1	162.63 (18)
C21—C22—H22	119.4	P2—Ru1—P1	83.97 (7)
C23—C22—H22	119.4	N1—Ru1—C27	120.6 (2)
C22—C23—C24	120.1 (7)	C35—Ru1—C27	38.6 (2)
C22—C23—H23	119.9	C34—Ru1—C27	61.5 (2)
C24—C23—H23	119.9	C33—Ru1—C27	61.3 (3)
C25—C24—C23	119.6 (8)	P2—Ru1—C27	156.39 (18)
C25—C24—H24	120.2	P1—Ru1—C27	101.4 (2)
C23—C24—H24	120.2	N1—Ru1—C32	95.3 (2)
C24—C25—C26	119.7 (7)	C35—Ru1—C32	62.8 (2)
C24—C25—H25	120.2	C34—Ru1—C32	61.3 (2)
C26—C25—H25	120.2	C33—Ru1—C32	36.7 (2)
C25—C26—C21	121.6 (6)	P2—Ru1—C32	147.10 (19)
C25—C26—H26	119.2	P1—Ru1—C32	128.82 (19)
C21—C26—H26	119.2	C27—Ru1—C32	36.0 (2)
